# Clinical value of dual detector spectral CT in fracture risk prediction in patients with type 2 diabetes mellitus: A retrospective cross-sectional study

**DOI:** 10.1097/MD.0000000000044347

**Published:** 2025-09-19

**Authors:** Fei Wang, Qin Wang, Lei Wu, Jin Liu, Minchao Xiong, Jun Chen, Yuxiang Wang

**Affiliations:** a Department of Medical Imaging, Ezhou Central Hospital, Ezhou, China.

**Keywords:** dual-layer spectral detector CT, type 2 diabetes mellitus, volumetric bone density

## Abstract

This retrospective cross-sectional study aimed to evaluate the clinical utility of dual-layer detector spectral CT (DLCT) in identifying bone mass abnormalities and predicting fracture risk among patients with type 2 diabetes mellitus (T2DM). Fifty patients with T2DM (28 males, 22 females; mean age, 65.21 ± 8.32 years) who underwent dual-energy X-ray absorptiometry (DXA), quantitative computed tomography (QCT), and DLCT imaging of the lumbar spine and hips between January 2023 and December 2024 were retrospectively analyzed. Areal bone mineral density and T-scores at L1–L2 and bilateral hips were obtained using DXA, while volumetric bone mineral density (vBMD) was assessed by QCT. DLCT virtual monoenergetic images at 40 and 70 keV were reconstructed to calculate the energy spectral attenuation slope (γ value, HU/keV). Bone mass status was classified as normal, low bone mass, or osteoporosis according to WHO and Chinese QCT criteria. Statistical analysis included Fisher exact test and Pearson correlation. The detection rates of abnormal bone mass by DXA, QCT, and DLCT were 35.7%, 60.7%, and 64.3% in males, and 45.5%, 68.2%, and 72.7% in females, respectively. QCT and DLCT had significantly higher detection rates than DXA (*P* < .05), with no significant difference between QCT and DLCT (*P* > .29). DLCT-derived γ values showed strong correlations with QCT-measured vBMD at L1, L2, and both hips (*r* = 0.921–0.984, all *P* < .05), with consistent findings in sex-stratified analyses (*r* ≥ 0.933, *P* < .05). DLCT-derived γ values are strongly correlated with QCT-derived vBMD and outperform DXA in detecting bone abnormalities in T2DM patients. DLCT offers a reliable, opportunistic tool for fracture risk assessment during routine CT imaging.

## 1. Introduction

Type 2 diabetes mellitus (T2DM) is one of the fastest-growing chronic metabolic diseases worldwide. According to the International Diabetes Federation, its global prevalence exceeded 537 million adults in 2021, with a projected annual growth rate of approximately 1%.^[1]^ In addition to cardiovascular, renal, and neurological complications, T2DM is increasingly recognized for its negative impact on bone health. Approximately 60% of diabetic patients exhibit some degree of skeletal impairment, with diabetic osteoporosis (DOP) affecting nearly 20% of individuals with T2DM.^[2]^

Studies have reported a significantly higher fracture incidence among T2DM patients compared to non-diabetics.^[3,4]^ These fractures are associated with increased disability and mortality, highlighting the urgent need for improved screening and risk assessment strategies in this population.

Currently, dual-energy X-ray absorptiometry (DXA) is the standard method for diagnosing osteoporosis.^[5]^ However, a phenomenon known as the “diabetic bone paradox” is often observed—T2DM patients may have normal or even elevated areal bone mineral density while remaining at high fracture risk.^[6,7]^ DXA’s inability to distinguish between cortical and trabecular bone, and its susceptibility to artifacts such as degenerative changes and vascular calcifications, limit its accuracy in this context.^[8]^

Quantitative computed tomography (QCT), including central QCT and high-resolution peripheral QCT, enables three-dimensional assessment of volumetric bone mineral density (vBMD) and provides superior sensitivity to trabecular changes.^[9,10]^ Nevertheless, its widespread clinical use is limited by cost, higher radiation exposure, and lack of standardization.^[11]^

Dual-layer detector spectral CT (DLCT), a recent innovation in spectral imaging, allows for simultaneous acquisition of high- and low-energy data using stacked detectors. This enables calculation of virtual monoenergetic images, electron density, and energy-dependent attenuation slopes, facilitating more accurate assessment of bone mineral content and microarchitecture.^[12–15]^

While prior studies have validated the use of DLCT for osteoporosis assessment in general populations,^[13,16]^ its clinical utility in T2DM patients—who may present with preserved bone mineral density (BMD) but structurally fragile bone—remains insufficiently explored. This study aims to evaluate the diagnostic performance of DLCT-derived γ values in comparison with DXA and QCT, and to determine its potential for opportunistic screening and fracture risk prediction in T2DM.

## 2. Methodology

### 2.1. Study design and population

This study was approved by the Ethics Committee of Ezhou Central Hospital (approval no. 2022-LL-027). This retrospective cross-sectional study included 50 patients with T2DM (28 males, 22 females; mean age 65.21 ± 8.32 years; mean disease duration 5.65 ± 2.89 years) who attended the Department of Endocrinology at our institution between January 2023 and December 2024 and underwent lumbar spine and hip imaging via DXA, QCT, and DLCT.

Inclusion criteria were as follows: diagnosis of T2DM according to the 2022 American Diabetes Association criteria; no anti-osteoporotic therapy within the 3 months prior to imaging; imaging quality sufficient to meet analysis requirements.

Exclusion criteria included: presence of secondary osteoporosis (e.g., hyperparathyroidism, long-term glucocorticoid therapy), history of lumbar or hip surgery, or metal implants; recent (within the past year) spinal or hip fracture; severe spinal deformities or tumor-related bone lesions.

### 2.2. Image acquisition and measurement

#### 2.2.1. DXA examination

Areal bone mineral density of lumbar vertebrae L1 and L2, as well as both hips, was measured using a GE Lunar Prodigy DXA system (GE Healthcare) with patients in the standard supine position. T-scores were automatically generated by the system.

#### 2.2.2. QCT examination

vBMD was assessed using Mindways QCT Pro software (Mindways Software Inc.). CT scans were performed with the following parameters: 120 kVp, automatic tube current modulation (100–200 mA), pitch of 0.6, and a slice thickness of 1 mm. A model 4 calibration phantom was included within the scanning field to ensure accurate calibration. During post-processing, vBMD (mg/cm³) was calculated within the central trabecular region of vertebrae L1 and L2, as well as in defined regions of interest (ROIs) in both hips.

#### 2.2.3. DLCT examination

A Philips IQon dual-layer spectral CT system (Philips Healthcare, Netherlands) was employed to perform both conventional and spectral imaging. Scanning parameters included 120 kVp, automatic tube current modulation, a pitch of 0.6, and a slice thickness of 1 mm. Raw spectral base image data were reconstructed to generate virtual monoenergetic images at 40 and 70 keV. CT attenuation values at 40 keV (CT_40_keV) and 70 keV (CT_70_keV) were measured at the same ROIs used for QCT analysis.

#### 2.2.4. ROI outlining and parameter calculation

**Figure 1.**
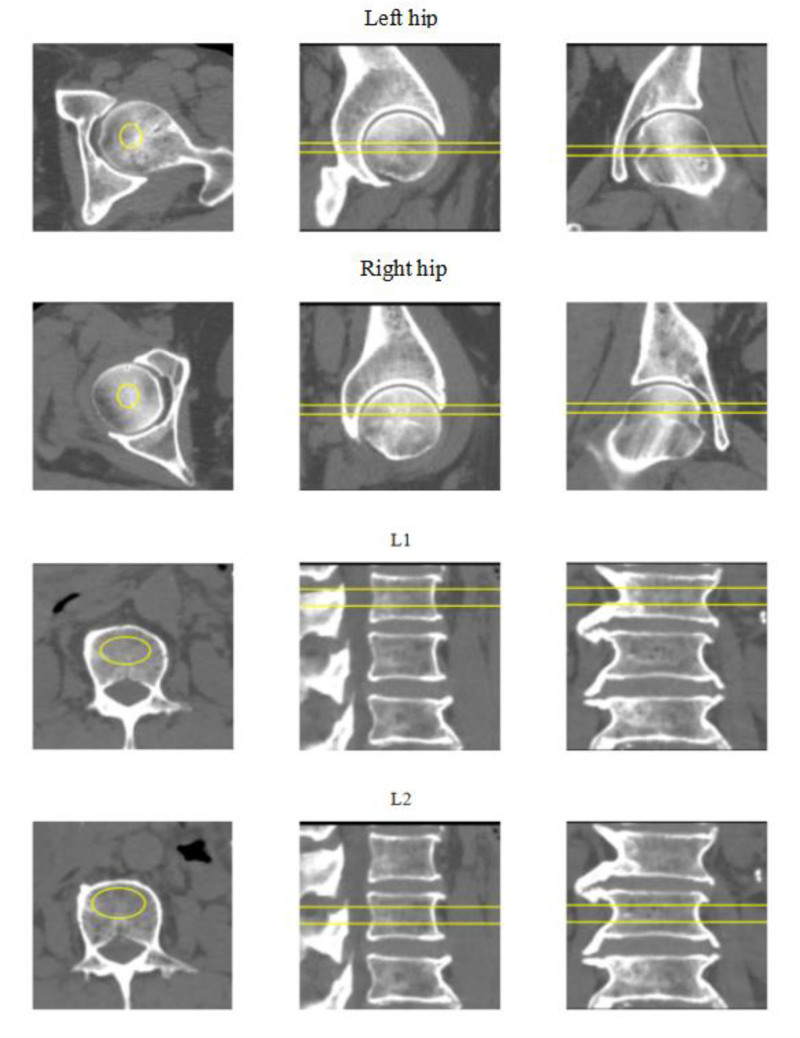
ROI outlining and parameter calculation. ROI = regions of interest.

ROI placement: Regions of interest were selected on axial images within the central trabecular bone of L1 and L2 vertebrae, as well as the bilateral femoral neck, carefully excluding cortical bone and vascular structures. Each ROI was measured 3 times, and the average value was recorded (Fig. 1).

Slope of the energy spectral attenuation curve (γ value): γ = (CT_40_keV − CT_70_keV) HU/(70 − 40) keV.

### 2.3. Diagnostic criteria and grouping

DXA classification (based on WHO criteria): T-score > –1.0 indicates normal bone mass; –2.5 < T-score ≤ –1.0 indicates low bone mass; T-score ≤ –2.5 indicates osteoporosis.

QCT classification (based on Chinese QCT guidelines): vBMD > 120 mg/cm^3^ is considered normal; 80 to 120 mg/cm^3^ indicates low bone mass; and vBMD < 80 mg/cm^3^ indicates osteoporosis.

For analysis, “abnormal bone mass” was defined as the combination of low bone mass and osteoporosis. The diagnostic performance of each imaging modality was compared based on this classification.

### 2.4. Statistical analysis

Statistical analysis was performed using SPSS version 26.0. Continuous variables were presented as mean ± standard deviation (x±s), while categorical variables were expressed as frequencies and percentages (%). Fisher exact test was employed to compare the diagnostic accuracy of DXA, QCT, and DLCT for identifying abnormal bone mass (P_1_: DXA vs QCT; P_2_: DXA vs DLCT; P_3_: QCT vs DLCT). Pearson correlation analysis was used to evaluate the relationship between QCT-derived vBMD and DLCT-derived γ-values, with correlation coefficients (*r*) and 95% confidence intervals (CIs) reported. Subgroup comparisons by sex were also conducted. A 2-tailed *P*-value < .05 was considered statistically significant.

## 3. Results

### 3.1. Baseline information of patients

A total of 50 patients with type 2 diabetes mellitus were enrolled in this study, comprising 28 males and 22 females. The overall mean age was 65.21 ± 8.32 years, with males averaging 67.46 ± 6.51 years and females 62.11 ± 4.87 years. The mean body mass index was 23.41 ± 2.65 kg/m^2^, with values of 23.97 ± 2.11 kg/m^2^ for males and 22.91 ± 1.32 kg/m^2^ for females. Mean glycated hemoglobin was 8.21 ± 2.33%, with males at 8.51 ± 1.77% and females at 7.91 ± 1.97%. The average duration of diabetes was 5.65 ± 2.89 years, 6.11 ± 1.54 years in males and 4.79 ± 1.85 years in females (see Table 1).

**Table 1:** Baseline information of patients.

Projects	Total (n = 50)	Female (n = 22)	Male (n = 28)
Age	65.21 ± 8.32	62.11 ± 4.87	67.46 ± 6.51
BMI	23.41 ± 2.65	22.91 ± 1.32	23.97 ± 2.11
Glycated hemoglobin	8.21 ± 2.33	7.91 ± 1.97	8.51 ± 1.77
Duration of illness	5.65 ± 2.89	4.79 ± 1.85	6.11 ± 1.54
DXA L1	‐0.87 ± 1.02	‐1.12 ± 1.31	‐0.57 ± 1.33
DXA L2	‐0.79 ± 1.21	‐1.19 ± 1.18	‐0.52 ± 1.45
DXA hip (left)	‐0.67 ± 1.23	‐0.91 ± 0.91	‐0.44 ± 1.01
DXA hip (right)	‐0.69 ± 1.21	‐0.88 ± 0.83	‐0.42 ± 1.13
QCT L1	97.06 ± 30.27	91.33 ± 24.11	102.54 ± 26.12
QCT L2	95.66 ± 31.17	89.43 ± 25.32	100.84 ± 27.38
QCT hip (left)	910.32 ± 119.09	899.45 ± 111.34	915.34 ± 110.76
QCT hip (right)	912.57 ± 118.75	903.32 ± 112.56	916.31 ± 108.56
DLCT L1	82.22 ± 25.43	80.21 ± 22.97	85.54 ± 22.03
DLCT L2	83.23 ± 28.23	80.88 ± 23.69	87.23 ± 22.69
DLCT hip (left)	843.34 ± 121.51	810.55 ± 101.77	867.21 ± 99.76
DLCT hip (right)	848.11 ± 129.02	803.76 ± 104.55	860.33 ± 103.22
DXA mean (lumbar)	‐0.83 ± 1.12	‐1.15 ± 1.43	‐0.55 ± 1.02
QCT mean (lumbar)	96.23 ± 30.89	90.35 ± 24.88	101.33 ± 26.79
DLCT mean (lumbar spine)	82.71 ± 26.19	80.55 ± 23.21	86.44 ± 22.14
DXA mean (hip)	‐0.68 ± 1.22	‐0.89 ± 0.86	‐0.43 ± 1.06
QCT mean (hip)	911.54 ± 118.85	901.38 ± 111.89	915.89 ± 109.65
DLCT mean (hip)	846.23 ± 125.67	807.21 ± 102.79	863.56 ± 101.28

DLCT = dual-layer detector spectral CT, DOP = diabetic osteoporosis, DXA = dual-energy X-ray absorptiometry, QCT = quantitative computed tomography.

### 3.2. Comparison of the accuracy of the methods in diagnosing bone mass abnormalities

The diagnostic accuracy of DXA, QCT, and DLCT for detecting abnormal bone mass (defined as low bone mass plus osteoporosis) was evaluated by sex.

In males (n = 28): DXA identified 18 cases (64.3%) as normal and 10 cases (35.7%) as abnormal. QCT identified 11 cases (39.3%) as normal and 17 cases (60.7%) as abnormal. DLCT identified 10 cases (35.7%) as normal and 18 cases (64.3%) as abnormal. Comparative analysis showed that both QCT and DLCT had significantly higher detection rates than DXA (DXA vs QCT, P_1_ = 0.03; DXA vs DLCT, P_2_ = 0.02), while there was no significant difference between QCT and DLCT (P_3_ = 0.358).

In females (n = 22):DXA identified 12 cases (54.5%) as normal and 10 cases (45.5%) as abnormal. QCT identified 7 cases (31.8%) as normal and 15 cases (68.2%) as abnormal. DLCT identified 6 cases (27.3%) as normal and 16 cases (72.7%) as abnormal. Similarly, QCT and DLCT outperformed DXA in detecting abnormal bone mass (DXA vs QCT, P_1_ = 0.04; DXA vs DLCT, P_2_ = 0.02), with no significant difference between QCT and DLCT (P_3_ = 0.298).

Sex-based comparison: No statistically significant differences were observed in diagnostic accuracy between males and females for DXA (*P* = .235), QCT (*P* = .189), or DLCT (*P* = .258) (see Table 2).

**Table 2:** Comparison of the accuracy of the methods in diagnosing bone mass abnormalities.

Gender	Numbers	DXA (n (%))		QCT (n (%))		DLCT (n (%))		*P*-value		
		Normal bone mass	Abnormal bone mass	Normal bone mass	Abnormal bone mass	Normal bone mass	Abnormal bone mass	*P*_1_	*P*_2_	*P*_3_
Male	28	18 (64.3%)	10 (35.7%)	11 (39.3%)	17 (60.7%)	10 (35.7%)	18 (64.3%)	.03	.02	.358
Female	22	12 (54.5%)	10 (45.5%)	7 (31.8%)	15 (68.2%)	6 (27.3%)	16 (72.7%)	.04	.02	.298
*P*		.235		.189		.258				

P1 = DXA compared with QCT, P2 = DXA compared with DLCT, P3 = QCT compared with DLCT.

DLCT = dual-layer detector spectral CT, DXA = dual-energy X-ray absorptiometry, QCT = quantitative computed tomography.

### 3.3. Correlation analysis between QCT vBMD and DLCT γ values

To evaluate the concordance between DLCT-derived γ values and vBMD measured by QCT, Pearson correlation analyses were conducted for vertebral bodies L1 and L2, as well as for both hips (see Table 3).

**Table 3:** Correlation analysis between QCT vBMD and DLCT γ values.

Clusters	Number of examples	QCT (mg/cm^3^)	γ-value (Hu/keV)	*r*	95% CI	*P*-value
Lumbar						
L1	50	97.06 ± 30.27	5.33 ± 1.18	0.921	0.897–0.954	<.05
L2	50	95.66 ± 31.17	5.31 ± 1.23	0.979	0.909–0.948	<.05
Totally	100	96.23 ± 30.89	5.32 ± 1.21	0.966	0.901–0.951	<.05
Hip						
Left	50	910.32 ± 119.09	37.01 ± 2.43	0.984	0.911–0.975	<.05
Right	50	912.57 ± 118.75	36.89 ± 2.87	0.936	0.929–0.968	<.05
Totally	100	911.54 ± 118.85	36.96 ± 2.61	0.954	0.920–0.971	<.05

DLCT = dual-layer detector spectral CT, QCT = quantitative computed tomography, vBMD = volumetric bone mineral density.

Lumbar spine measurements: L1 vertebra (n = 50): The mean QCT-derived vBMD was 97.06 ± 30.27 mg/cm^3^, and the corresponding DLCT γ-value was 5.33 ± 1.18 HU/keV. A strong positive correlation was observed between the 2 measures (*r* = 0.921, 95% CI: 0.897–0.954, *P* < .05). L2 vertebra (n = 50): The mean QCT vBMD was 95.66 ± 31.17 mg/cm^3^, with a corresponding DLCT γ-value of 5.31 ± 1.23 HU/keV. The correlation coefficient was *r* = 0.979 (95% CI: 0.909–0.948, *P* < .05). Overall lumbar spine (n = 100): The average QCT vBMD across L1 and L2 was 96.23 ± 30.89 mg/cm^3^, and the mean DLCT γ-value was 5.32 ± 1.21 HU/keV, showing a strong positive correlation (*r* = 0.966, 95% CI: 0.901–0.951, *P* < .05).

Hip measurements: Left hip (n = 50): The mean QCT-derived vBMD was 910.32 ± 119.09 mg/cm^3^, and the corresponding DLCT γ-value was 37.01 ± 2.43 HU/keV. A strong positive correlation was observed (*r* = 0.984, 95% CI: 0.911–0.975, *P* < .05). Right hip (n = 50): The mean QCT vBMD was 912.57 ± 118.75 mg/cm^3^, with a γ-value of 36.89 ± 2.87 HU/keV. The correlation coefficient was *r* = 0.936 (95% CI: 0.929–0.968, *P* < .05). Overall hip (n = 100): The combined mean QCT vBMD was 911.54 ± 118.85 mg/cm^3^, and the average DLCT γ-value was 36.96 ± 2.61 HU/keV, with a strong correlation (95% CI: 0.920–0.971, *P* < .05). These findings indicate that the DLCT-derived energy spectral attenuation slopes (γ-values) are highly consistent with QCT-measured volumetric BMD at both the lumbar spine and hip (*r* range: 0.921–0.984, all *P* < .05), supporting the potential of DLCT γ-values as reliable surrogate or complementary indicators of vBMD.

### 3.4. Correlation analysis stratified by gender

To further evaluate the agreement between DLCT-derived γ-values and QCT-measured vBMD across sexes, Pearson correlation analyses were conducted separately for male and female patients.

Lumbar spine: Males (n = 28): The mean QCT vBMD was 101.33 ± 26.79 mg/cm^3^, and the corresponding DLCT γ-value was 5.51 ± 1.21 HU/keV, showing a strong positive correlation (*r* = 0.945, 95% CI: 0.921–0.961, *P* < .05). Females (n = 22): The mean QCT vBMD was 90.35 ± 24.88 mg/cm^3^, with a DLCT γ-value of 5.13 ± 1.25 HU/keV, also demonstrating a strong correlation (*r* = 0.934, 95% CI: 0.914–0.955, *P* < .05).

Hip: Males (n = 28): QCT vBMD was 915.89 ± 109.65 mg/cm^3^, and the DLCT γ-value was 37.25 ± 2.35 HU/keV, with a correlation coefficient of *r* = 0.933 (95% CI: 0.907–0.961, *P* < .05). Females (n = 22): QCT vBMD measured 901.38 ± 111.89 mg/cm^3^, and the γ-value was 36.47 ± 2.41 HU/keV, with a correlation of *r* = 0.952 (95% CI: 0.927–0.974, *P* < .05).

These findings confirm that DLCT γ-values are strongly correlated with QCT-derived vBMD at both lumbar spine and hip sites in both sexes, with correlation coefficients ranging from 0.933 to 0.952 (all *P* < .05) (see Table 4).

**Table 4:** Correlation analysis stratified by gender.

Clusters	Number of examples	QCT (mg/cm^3^)	γ-value (Hu/keV)	*r*	95% CI	*P*-value
Lumbar						
Male	28	101.33 ± 26.79	5.51 ± 1.21	0.945	0.921–0.961	<.05
Female	22	90.35 ± 24.88	5.13 ± 1.25	0.934	0.914–0.955	<.05
Hip						
Male	28	915.89 ± 109.65	37.25 ± 2.35	0.933	0.907–0.961	<.05
Female	22	901.38 ± 111.89	36.47 ± 2.41	0.952	0.927–0.974	<.05

QCT = quantitative computed tomography.

## 4. Discussion

In this study, we systematically evaluated—for the first time—the clinical utility of the energy spectral attenuation slope (γ-value) derived from DLCT for assessing bone mass and predicting fracture risk in patients with T2DM.

The key findings were as follows: DLCT γ-values demonstrated strong correlations with vBMD of the lumbar spine and hip as measured by QCT (*r* range: 0.921–0.984, *P* < .05), with consistent results across sex-stratified analyses; DLCT showed significantly greater diagnostic sensitivity for detecting abnormal bone mass (defined as low bone mass plus osteoporosis) compared to conventional DXA, and its diagnostic performance was comparable to that of QCT; No significant sex-based differences were observed in the diagnostic accuracy of DXA, QCT, or DLCT, indicating that DLCT is broadly applicable to both male and female T2DM populations.

Although DXA remains the clinical “gold standard” for evaluating BMD, it frequently demonstrates the “diabetic bone paradox” in patients with T2DM—that is, normal or elevated BMD despite an increased risk of fracture.^[17]^ In this study, the diagnostic performance of DXA in detecting abnormal bone mass in T2DM patients was limited, with detection rates of only 35.7% in males and 45.5% in females. These rates were significantly lower than those observed with QCT (60.7% and 68.2%, respectively) and DLCT (64.3% and 72.7%, respectively). In contrast, DLCT-derived γ-values not only showed strong correlation with QCT-measured volumetric BMD, but also outperformed DXA in detecting bone loss and osteoporosis. These findings suggest that DLCT offers enhanced sensitivity in evaluating bone quality in T2DM patients and may help reduce false-negative diagnoses in this population.

Several studies have validated the use of DLCT for assessing vertebral BMD in healthy populations using the slope of the energy spectral attenuation curve, with reported correlation coefficients exceeding 0.93.^[17]^ Wang MM et al further confirmed that DLCT-derived vBMD is highly consistent with QCT measurements and demonstrates superior accuracy compared to DXA in patients undergoing spinal surgery.^[18]^ Building upon these findings, the present study focuses specifically on individuals with T2DM, highlighting the unique advantages of DLCT in screening for diabetic osteoporosis. Additionally, Zhang Yu et al reported similar conclusions when comparing QCT and DXA in postmenopausal women, supporting the notion that volumetric BMD offers greater diagnostic value than areal BMD.^[19]^ Our results further demonstrate that DLCT γ-values not only quantify BMD but also provide insights into bone microarchitecture and material composition, offering a more comprehensive and intuitive reflection of diabetes-related skeletal alterations.^[20]^

In patients with T2DM, bone microstructure and mechanical integrity are often compromised due to the accumulation of advanced glycation end-products, abnormal collagen cross-linking, and reduced bone matrix quality. DXA, which cannot differentiate between cortical and trabecular bone, is also limited in its ability to detect alterations in bone matrix composition. DLCT, by simultaneously capturing CT data at both 40 and 70 keV energy levels, allows for calculation of the energy spectral attenuation slope (γ-value). This parameter reflects differences in bone mineral concentration and indirectly provides information on calcium salt distribution and trabecular integrity within the bone matrix.^[21]^ A higher γ-value indicates greater vBMD and increased bone calcium content. In this study, we observed a strong correlation between DLCT-derived γ-values and QCT-measured vBMD at both the lumbar spine and hip in T2DM patients. These findings suggest that the DLCT γ-value may serve as a reliable surrogate or complementary marker for vBMD, offering a novel “opportunistic” approach for early detection and fracture risk assessment in DOP.^[22]^

With the rising global prevalence of T2DM, the associated risk of osteoporotic fractures is also increasing; however, the coverage of conventional DXA screening remains inadequate.^[23]^ DLCT offers a practical advantage by enabling extraction of γ-values from routine lumbar spine and hip CT scans without additional radiation exposure or cost, making it more feasible for widespread clinical use compared to QCT. In routine clinical practice, diabetic patients frequently undergo abdominal or spinal CT imaging. DLCT allows for simultaneous assessment of bone mineral density and fracture risk prediction during these examinations, achieving a “one scan, multiple outcomes” approach.^[24]^ Looking ahead, integrating DLCT-derived parameters with clinical tools such as FRAX scores and biochemical markers (e.g., osteocalcin and advanced glycation end-products) may enable the development of a comprehensive, multidimensional fracture risk prediction model—enhancing personalized prevention and management strategies for skeletal complications in T2DM.^[25]^

The strengths of this study include: comprehensive evaluation of 2 high-risk skeletal sites—the lumbar spine and hip—with complete and detailed data; direct assessment of the clinical applicability of DLCT through a comparative analysis involving DXA, QCT, and DLCT within the same patient cohort; and consistent validation of results across both sexes, enhancing the generalizability of the findings. However, several limitations should be noted. First, the study was a single-center retrospective analysis with a relatively small sample size, potentially introducing selection bias. Second, no longitudinal follow-up data on actual fracture outcomes were included, and the relationship between DLCT γ-values and incident fractures requires further validation in prospective cohort studies. Third, additional spectral parameters provided by DLCT—such as electron density and calcium suppression imaging—were not explored for their potential additive value in bone quality assessment. Lastly, manual ROI delineation may introduce subjectivity; therefore, the development of automated AI-based analysis workflows is warranted to enhance reproducibility and efficiency.

In the future, multicenter, large-scale prospective studies are warranted to refine the clinical diagnostic thresholds and fracture risk prediction models based on DLCT γ-values. Additionally, integrating emerging imaging modalities such as high-resolution peripheral QCT and MRI T2 mapping may further elucidate the comprehensive value of DLCT in evaluating bone microarchitecture and matrix quality. Stratified analyses should also be conducted to assess the sensitivity and specificity of DLCT γ-values across diverse T2DM subpopulations—for example, comparing early-onset versus late-onset cases, and patients with versus without diabetic nephropathy. Furthermore, investigating the correlations between DLCT-derived spectral multiparameters (e.g., electron density, virtual monoenergetic CT values, and relative spectral response) and biochemical bone turnover markers may enable a more holistic assessment of bone quality, bridging imaging findings with molecular-level insights.^[26]^

This study demonstrated that DLCT-derived γ-values can accurately quantify vBMD of the lumbar spine and hip in patients with T2DM. DLCT outperformed DXA and showed diagnostic performance comparable to QCT in detecting bone abnormalities, highlighting its potential as a valuable tool for early screening and fracture risk prediction in DOP. The ability of DLCT to assess bone quality directly from routine CT images—without additional radiation or cost—positions it as a practical and scalable approach for diabetes-related bone health monitoring. DLCT is poised to play a pivotal role in the comprehensive management of skeletal health in T2DM, offering imaging-based support for precision prevention and targeted intervention strategies.

## Author contributions

**Conceptualization:** Fei Wang, Yuxiang Wang.

**Data curation:** Fei Wang, Yuxiang Wang.

**Formal analysis:** Fei Wang.

**Funding acquisition:** Yuxiang Wang.

**Investigation:** Fei Wang, Yuxiang Wang.

**Methodology:** Fei Wang, Yuxiang Wang.

**Software:** Fei Wang.

**Supervision:** Fei Wang, Yuxiang Wang.

**Validation:** Fei Wang.

**Visualization:** Yuxiang Wang.

**Writing – original draft:** Fei Wang, Yuxiang Wang.

**Writing – review & editing:** Fei Wang, Yuxiang Wang.
